# Core outcomes set for studies on primary prevention of preterm birth

**DOI:** 10.1186/1745-6215-16-S1-P11

**Published:** 2015-05-29

**Authors:** Janneke van‘t Hooft, James M N Duffy, George R Saade, Zarko Alfirevic, S Meher, Ben Willem J Mol, Khalid S Khan

**Affiliations:** 1 Department of Obstetrics and Gynecology, Academical Medical Center, 1105DE Amsterdam, The Netherlands; 2 Nuffield Department of Primary Care Health Sciences, University of Oxford, Oxford, OX1 2JD, UK; 3 University of Texas Medical Branch, 301 University Boulevard, Galveston, TX 77555, United States; 4 Department of Women and Children’s Health, Institute of Translational Medicine, University of Liverpool, Liverpool, UK; 5 The Robinson Research Institute, School of Paediatrics and Reproductive Health, University of Adelaide, 5000 SA Australia; 6 Women's Health Research Unit, The Blizard Institute, Barts and the London School of Medicine and Dentistry, London, E1 2AB, UK

## Background

Trials and evaluations of interventions for preterm birth prevention have reported many different outcomes resulting in an inability to synthesise results across studies. Our objective was to produce a minimum set of important and critical outcomes (core outcome set) for studies, reviews, evaluations and guidelines on primary prevention of preterm birth.

## Materials and methods

Between May and November 2014 we went through the process of identification and selection of outcomes. All possible outcomes were drawn from systematic reviews, interviews and questionnaires. From this initial list, a selection process was performed using an online 2-round Delphic survey and a consultation meeting. Target stakeholders were approached to contribute in this selection (parents, midwives, obstetricians, neonatologists and methodologists) through purposive sampling in relevant networks: patient organisations, midwife networks, Global Obstetrics Network (GONet), the Cochrane collaboration and journal editors from the Core Outcomes in Women’s health (CROWN) initiative.

## Results

From an initial list of 249 items, 29 outcomes were identified for the process of consensus in the Delphi survey. A total of 228 participants were approached for the Delphi survey of whom 194 (86%) completed the first survey and 174 (89%) the second survey. Responders of both surveys represented all stakeholder groups: parents (n=25), midwives, (n=25), obstetricians (n=54), neonatologists (n=34), methodologists (n=34) and industry (n=2) from 11 low and 16 high resourced countries. The following eleven outcomes were selected as being ‘critical outcomes’ in the Delphi survey and one more was included after the consultation meeting: [1] maternal mortality; [2] maternal infection or inflammation; [3] gestation age at delivery; [4] offspring mortality; [5] birth weight; [6] offspring early developmental morbidity; [7] offspring late neurodevelopmental morbidity; [8] offspring gastro-intestinal morbidity; [9] offspring infection; [10] offspring respiratory morbidity; [11] prelabor rupture of membranes; [12] harm.

## Conclusions

We developed a core outcome set for studies on primary prevention of preterm birth. We encourage researchers to start to collect all outcomes that are in this core outcome set.

## Trial Registration

COMET Registration Number: 603.

http://www.comet-initiative.org/studies/details/603.

